# Exploring Teacher Awareness of Artificial Intelligence in Education: A Case Study from Northern Cyprus

**DOI:** 10.3390/ejihpe14080156

**Published:** 2024-08-12

**Authors:** Ahmet Güneyli, Nazım Serkan Burgul, Sonay Dericioğlu, Nazan Cenkova, Sinem Becan, Şeyma Elif Şimşek, Hüseyin Güneralp

**Affiliations:** 1 Faculty of Education, European University of Lefke, Lefke 99728, Northern Cyprus, Mersin 10, Türkiye; 2 Faculty of Sports Sciences, Near East University, Lefkoşa 99138, Northern Cyprus, Mersin 10, Türkiye; nazim.burgul@neu.edu.tr (N.S.B.); sinem.becan@neu.edu.tr (S.B.); elif.simsek@neu.edu.tr (Ş.E.Ş.); huseyin.guneralp@neu.edu.tr (H.G.); 3 Faculty of Education, University of Mediterranean Karpasia, Lefkoşa 99010, Northern Cyprus, Mersin 10, Türkiye; sonay.dericioglu@akun.edu.tr; 4 Atatürk Teacher Training Academy, Lefkoşa 99010, Northern Cyprus, Mersin 10, Türkiye; nazan.cenkova@aoa.edu.tr

**Keywords:** artificial intelligence in education, teacher awareness, AI application in teaching, professional development, Northern Cyprus education

## Abstract

This study investigates the level of awareness among teachers regarding the use of artificial intelligence (AI) in education, focusing on whether this awareness varies according to socio-demographic characteristics, access to technology, and specific knowledge and beliefs about AI. Conducted in Northern Cyprus during the 2023–2024 academic year, this study employed a survey model with purposive and snowball sampling methods, involving 164 teachers. Teachers at different levels, namely, primary school, secondary school, high school, and university, were included in this study. The “Artificial Intelligence Awareness Scale”, developed by Ferikoğlu and Akgün (2022), was used to measure AI awareness. Data normality was verified through skewness and kurtosis values, allowing for parametric statistical tests such as t-tests, one-way ANOVA, logistic regression, and chi-square analysis. This study explored the distribution of AI use across different school types and educational levels and assessed the impact of sub-dimensions of AI awareness on its application in teaching. Findings revealed no significant influence of teacher demographics (age, gender, education level, type of school, institution level, and monthly income) on AI awareness. However, usage patterns indicated that university lecturers were more likely to incorporate AI in their teaching, followed by primary and high school teachers, with secondary school teachers using it the least. A Multilayer Neural Network Analysis identified practical knowledge as the most critical factor influencing the use of AI in teaching (importance weight of 0.450), followed by beliefs and attitudes (0.298), relatability (0.148), and theoretical knowledge (0.104). These results highlight the importance of practical knowledge for fostering AI integration in educational practices, underscoring significant implications for teacher training and professional development programs.

## 1. Introduction

According to Edward Fredkin, a developer and recognized authority in the field of artificial intelligence, “There are three great events in history. The first is the creation of the universe. The second is the emergence of life. The third is the emergence of artificial intelligence” [1]. This statement suggests that the potential of artificial intelligence and its future impact are far beyond what we currently envision [2]. It is believed that this rapidly advancing technology will significantly enhance education from multiple perspectives and create greater momentum for overcoming the challenges encountered in the teaching process [3,4,5].

With advancements in artificial intelligence and technology, it has become possible to deliver education anytime and anywhere [6]. Additionally, the integration of artificial intelligence (AI) into education systems now offers options such as free choice, personalized learning, and project-based learning. Today, artificial intelligence systems are primarily used in education for distance learning, online learning, virtual reality, and augmented reality. The emergence of these systems has not only changed the type of individual that the education system aims to develop but also transformed the functioning of education itself [7,8]. Educational materials and software equipped with artificial intelligence offer capabilities such as thinking, abstracting, learning, adapting to new situations, and facilitating interaction, akin to human abilities [9]. However, it is important to critically assess these technologies, as AI is not a universal solution. While AI’s role in education, especially in active learning, is expanding and complementing other learning methods, it is essential to approach its benefits with caution. The body of research on this topic is growing daily, reflecting both the potential and the need for careful evaluation of AI in educational settings [10,11].

The unique factors that differentiate artificial intelligence from other educational technologies include the ability to match educational content to individual student needs, communicate with and respond to students, model a student’s learning process, decide what information to provide based on a student’s past performance, and make decisions about a student’s level of understanding and educational progression. The potential of artificial intelligence to transform education is expected to manifest increasingly in four key areas: (i) AI can provide personalized and effective support to students throughout the learning process, (ii) testing and assessment can gain a new dimension for both students and educators through AI, (iii) differentiated and personalized learning can be used more effectively and widely, and (iv) feedback, a critical component of education, can be automated by AI to meet students’ needs [3,12].

The rapid integration of artificial intelligence (AI) technology into educational environments underscores the critical need for educators to be prepared for technological change. As teachers gain knowledge and skills in AI, they can integrate AI into their lesson plans and customize and enrich their students’ learning experiences. In addition, it becomes easier to perform administrative tasks in education. Considering the fact that each student learns individually, artificial intelligence can be used to determine the learning needs of students [13]. By using artificial intelligence during the education process, students’ development and performance can also be monitored. Following this, teaching strategies are revised as a result of students’ performances and educational failure can be reduced. This data-based artificial intelligence support in education enables target-oriented and efficient education [14]. The demand for specialization in artificial intelligence in education is increasing day by day. The knowledge and skills required to be acquired regarding artificial intelligence are included in curriculums. The main purpose is; to prepare students for the future, ensure their career development, and increase the competitiveness of individuals in the technology-equipped labor market [15,16].

Artificial intelligence also has the opportunity to increase student engagement in education. Teachers who are aware of this contribution tend to use artificial intelligence in education based on the different learning styles and speeds of each student [17,18]. Administrative tasks such as grading and tracking students’ attendance status are carried out using artificial intelligence. This leaves more time for teacher–student interaction in education. Reducing the administrative burden on teachers can also positively affect classroom management [19]. Moreover, AI can provide real-time assistance to students through chatbots and virtual assistants, offering immediate help with assignments, answering queries, and providing feedback. Teachers who are familiar with these technologies can incorporate them into their lesson plans to provide continuous support, even after school hours [20,21]. Understanding AI also encourages educators to explore and implement cutting-edge pedagogies, such as flipped classrooms and gamified learning environments. These techniques create a more dynamic and participatory learning environment [22,23].

The background of this study on teacher awareness of artificial intelligence (AI) in education highlights the rapid advancements in AI technology and its significant potential to transform educational practices. AI’s capabilities, such as personalized learning, real-time feedback, and automation of administrative tasks, present substantial opportunities for enhancing educational outcomes. Artificial intelligence holds significant potential to enhance learning quality and efficiency, but several challenges must be addressed to achieve its optimal and equitable benefits. A thoughtful and inclusive strategy is essential to ensure AI supports educational objectives; otherwise, artificial intelligence may negatively impact students’ independence. Students may often resort to artificial intelligence to solve tasks that are particularly challenging and require active participation, and this may lead to artificial intelligence addiction, which can damage students’ critical thinking skills the most. In addition, social skills may be damaged, and interaction and cooperation may decrease during the learning–teaching process. The use of artificial intelligence in administrative tasks may lead to privacy and transparency issues. The possibility of ethical problems increases. Since not every school, teacher, or student has equal access to artificial intelligence technologies and infrastructure opportunities, inequality in education and digital divides may occur. The use of artificial intelligence in education may result in high costs, and technical support, software, etc. problems may increase. Finally, teachers are required to undergo in-service training. Expecting teachers to use artificial intelligence in their lessons without complete knowledge and skill levels may lead to significant problems [24]. As a result, in order to use artificial intelligence in education, the first step is to prepare educators and raise their awareness. The increase in demand for artificial intelligence training with adaptation to developments in educational technologies is seen as a result of this necessity [2,5,6].

The number of artificial intelligence studies has increased greatly today. Conducting a study on artificial intelligence in Northern Cyprus as a developing country can contribute to the international literature. The results of this research obtained from Cyprus can be compared with the research findings in developed countries. Finding out which socio-demographic variables affect teachers’ awareness of artificial intelligence is the starting point of this research. Socio-demographic variables such as gender, age, and education level were taken as the basis of the research. This study focused on whether the mentioned variables affected teachers’ application situations and beliefs regarding artificial intelligence [3,7,9]. In this study, a robust methodological approach was adopted using different parametric statistical techniques. For example, it is aimed at clarifying the critical factors in the use of artificial intelligence in education with Multilayer Neural Network Analysis [25]. The innovative aspect of this study is that different aspects of artificial intelligence, such as practical and theoretical knowledge, are considered and evaluated together in the same study. Obtaining data from a geographically different region is also necessary to contribute to the research literature. Methodologically, originality was created in the context of both the selected socio-demographic variables and the statistical techniques used. In this way, artificial intelligence education policies can be improved and teacher training programs can be developed. This issue is important in the context of exploring the transformative potential of artificial intelligence in education and implementing the use of artificial intelligence in education.

## 2. Theoretical Framework

This study emphasizes the value of practical AI training and professional development by examining instructors’ understanding of AI using the Technology Acceptance Model (TAM) and the Diffusion of Innovation Theory. It emphasizes that, as the TAM, which focuses on perceived ease of use and advantages highlights, individual attitudes and expectations are crucial to the adoption of new technologies. This study supports “learning by doing” as a means of enhancing AI integration in education, which is in line with constructivist learning theories and Bloom’s Taxonomy. This study indicates that focused educational policy and training can greatly improve the application of AI in teaching practices, thereby boosting educational outcomes, by addressing practical skills and creating favorable attitudes [26,27,28,29,30].

The Technology Acceptance Model (TAM), Rogers’ Diffusion of Innovation Theory, and Constructivist Learning/Learning by Doing Theory are among the many interesting intersections where artificial intelligence (AI) meets these frameworks. Davis developed a framework in 1989 called the Technology Acceptance Model to explain how people come to accept and use technology. It makes the argument that people’s views toward adopting technology are greatly influenced by their perceptions of its perceived usefulness and simplicity of use [31]. AI is a good fit for TAM because it can increase productivity and user experience through automation, tailored recommendations, and intelligent decision-making. Research indicates that AI’s acceptability and integration in a variety of industries, including healthcare and education, are driven by perceptions of its value in enhancing work performance and its simplicity in streamlining complicated operations.

Everett Rogers created the Diffusion of Innovation Theory in 1962, which explains how ideas are shared and grow over time among people in a social system. Five groups of adopters are identified by the theory: laggards, innovators, early adopters, early majority, and late majority [32]. AI is a transformational technology that spreads according to this method. While the early majority and late majority adopt AI as technology becomes more widely available and its advantages become more obvious, innovators and early adopters in tech-savvy industries swiftly use AI to gain a competitive advantage. For instance, the adoption of AI in education has demonstrated an increasing spread, matching Rogers’ stages.

According to Piaget and Vygotsky’s constructivist learning theory, learning is an active, constructive process in which students build new concepts using what they already know and have learned [33]. AI can support this method of instruction by offering dynamic, flexible learning settings that adjust to the demands of each unique student. As per constructivist principles, students participate in ‘learning by doing’ with the help of AI-driven customized learning systems. Artificial intelligence (AI) systems may replicate real-world situations, offering relevant, first-hand experiences that support theoretical understanding. Through the promotion of deeper comprehension and information retention through experiential learning, these applications show how AI can enhance constructivist pedagogy.

In conclusion, there is a significant connection between AI and these theoretical frameworks. According to TAM, AI’s capacity to boost perceived utility and usability encourages adoption. Its trend of acceptance is similar to the diffusion process that Rogers described, and its use in educational technologies is a prime example of constructivist learning and “learning by doing” These linkages demonstrate the important contribution artificial intelligence (AI) makes to the advancement of technology in a variety of domains, both theoretically and practically.

### 2.1. Artificial Intelligence in Education

The UNESCO report [34] outlines the status of AI curricula implementation in K-12 education across various countries. AI curricula have been endorsed and implemented in Armenia, Austria, Belgium, China, India, Kuwait, Portugal, Qatar, Serbia, South Korea, and the United Arab Emirates. AI curricula are in development in Bulgaria, Germany, Jordan, Saudi Arabia, and Serbia. Serbia has both implemented and is developing AI curriculum. For primary school, AI curricula have been implemented in all countries except Armenia, Austria, Belgium, India, Serbia, South Korea, and Jordan. For middle school, AI curricula have been implemented in all countries except Austria, Belgium, and South Korea. For high school, AI curricula have been implemented in all countries except Kuwait. The UNESCO report profiles the most emphasized topic areas in K-12 AI curricula. AI foundations are highlighted in 41% of the curricula, with specific focus on ‘algorithms and programming’ at 18%, ‘data literacy’ at 12%, and ‘contextual problem-solving’ at 11%. Understanding, using, and developing AI is emphasized in 25% of the curricula, with ‘AI technologies’ at 14%, ‘developing AI technologies’ at 9%, and ‘AI techniques’ at 2%. Ethics and social impact are covered in 24% of the curricula, with the ‘application of AI to other domains’ at 12%, ‘ethics of AI’ at 7%, and ‘social implications of AI’ at 5%. An additional 10% of the topic areas are unspecified.

Survey respondents reported learning hours for four educational levels: early primary (K-2), late primary (3–6), middle school (7–9), and senior/high school (10–12). Curriculum hours varied widely, from 2 to 924 h across grades. Qatar’s Computing and Information Technology and Belgium’s IT Repository were outliers, averaging over 200 h per year. The average was 58 h per year, double the median of 21 h, indicating many curricula require minimal AI study. Five of 22 curricula needed less than 5 h of AI study per year, while five required 150 h or more. Most curricula targeted higher grade levels. Specific time allocations were as follows: K-2 often had AI integrated into other subjects, except for Qatar’s 100-h program; grades 3–6 averaged 156 h; grades 7–9 averaged 109 h; and grades 10–12 averaged 153.5 h. Hours per grade were stable for K-9 (33.3 to 39 h) but increased to 51.2 h in high school [34].

The 2023 Artificial Intelligence Index Report [35] highlights trends in higher education. AI specialization in new computer science Ph.D. graduates from U.S. universities rose to 19.1% in 2021, up from 14.9% in 2020 and 10.2% in 2010. More AI Ph.D.s are moving to industry, with 65.4% in 2021, compared to 40.9% in 2011. The total number of new North American computer science, computer engineering, and information faculty hires decreased from 733 in 2012 to 710 in 2021, with tenure-track hires peaking at 422 in 2019 before dropping to 324 in 2021. In 2021, 78.7% of new AI Ph.D.s were male, showing a persistent gender imbalance despite a slight increase in female Ph.D.s. The U.S. and China led cross-country collaborations in AI publications from 2010 to 2021, but the growth rate of these collaborations has slowed recently. AI research publications have more than doubled since 2010, focusing on pattern recognition, machine learning, and computer vision. China leads in total AI publications, while the U.S. leads in AI conference and repository citations, though this lead is diminishing. In 2022, 54% of the world’s large language and multimodal models were produced by American institutions.

In order to increase productivity and streamline repetitive work, schools are progressively incorporating AI into their operations [36]. For example, the Central Board of Secondary Education (CBSE) in India declared that artificial intelligence (AI) would be offered as an elective in over 22,000 of its member schools [34]. Schools are using AI more frequently as they realize how much it can improve student learning [37]. Some nations, like China, are incorporating AI into their high school curricula, emphasizing programming, machine learning, and decision-making. However, its use at the primary level is less common [38]. There is an increasing interest in AI education, as seen by the workshops, courses, and projects being conducted in several countries to examine AI teaching-learning processes [39].

In conclusion, there are many different regulations pertaining to students’ use of AI, all of which seek to strike a balance between the development of technology, moral issues, and fair access to education. To optimize the advantages of AI in education, these policies call for thorough frameworks, institutional rules, and ongoing training for teachers and students. Different nations tackle AI policies in education in different ways. For instance, in Vietnam, legislative regulations pertaining to AI and e-learning are being examined to adjust to how breakthroughs in technology are affecting educational practices [40]. Comparably, to enhance education and learning using AI, the Indonesian government is concentrating on inclusive policies [41].

### 2.2. Artificial Intelligence and Teachers

Teachers must play a central role in the effective use of artificial intelligence (AI) in the classroom, acting as decision-makers regarding when and how to use AI tools. Additionally, AI tools and the data they provide can help teachers optimize the use of various resources. The increasing influence of AI-based tools indicates a shift in the role of the teacher, transforming them into facilitators who enhance learning experiences through technology [42,43,44]. AI supports education by assisting teachers and providing meaningful learning experiences [10]. From the teachers’ perspective, the benefits of AI include offering effective teaching methods, facilitating gamified teaching, and assisting in the preparation of curricula, lesson plans, and activities, as well as enhancing the understanding, assessment, and analysis of students [45]. Research by Osetskyi et al. [46] further highlights the advantages of AI for teachers, such as ease of student management, automation of tasks and content creation, continuous improvement, objective assessments, rapid and comprehensive feedback, performance monitoring, and support in developing teaching skills. As Aşık and colleagues [47] have noted, AI can significantly reduce teachers’ workload, enabling them to use their valuable time more efficiently.

AI-enabled materials foster the development of current and relevant content, reduce learning times, provide dynamic content that deviates from routine methods, and maintain student interest. By automating tasks such as preparing and marking assignments and exams, teachers can dedicate more time to communicating and interacting with students. Automated systems offer rapid feedback, active correction, and precise performance guidance [48]. International studies support the Turkish literature in recognizing how AI can benefit educators [49,50,51].

Teachers’ use of AI varies according to recent studies. About 83.4% of teachers acknowledge that AI is helpful for their professional development in education [52]. The percentage of teachers actively using AI in their teaching practice can be considered moderate. At Sana’a University, there is a moderate level of AI use among teachers, with a high awareness of its importance and the barriers to its use [53]. Despite the recognition of the benefits of AI, there are significant concerns among educators about the role of AI. Many believe that human teachers have unique qualities that make them irreplaceable, suggesting a cautious approach to the widespread adoption of AI in education [54]. Overall, while a significant proportion of teachers recognize the benefits of AI and find it helpful for professional development, the actual percentage of teachers actively using AI in their teaching practice is likely to be moderate. This is influenced by factors such as awareness, perceived benefits, challenges, and concerns about AI replacing human teachers.

Despite rapid advancements in AI, it is crucial not to rely solely on technology in education. Technologies should be viewed as supportive elements rather than replacements, ensuring the human aspect is not overlooked in educational processes [45]. It is also important to address the potential risks and concerns of AI in education, such as the need for effective regulations on ethics, data privacy, and personal information protection to build confidence among administrators, teachers, students, and parents [55]. Moreover, the potential for AI to replace professional roles, which could lead to job losses and induce stress among teachers, requires careful consideration. Predictions on how the role of teachers will evolve with AI development must include the human factor to prevent adverse effects on teacher productivity [48]. Çetin and Aktaş [56] have demonstrated through qualitative research that the current capabilities of AI alone are insufficient to replace a teacher in the classroom.

In the Turkish literature on AI use in education, studies reveal teachers’ perspectives on various aspects, including potential applications of AI in the teaching process, how AI can enhance student learning and success, recommendations for integrating AI into teaching (classroom management, teaching methods, assessment, and evaluation), challenges or concerns in AI-enhanced education, and the ethical and security aspects of AI-based learning tools [57,58,59,60,61,62,63].

The integration of artificial intelligence (AI) in education is reshaping the roles of teachers, necessitating a paradigm shift in their professional responsibilities and the acceptance of new technologies. As AI tools become more prevalent, teachers are evolving from traditional information providers to facilitators of technology-enhanced learning experiences. This transformation is supported by research indicating that teachers must navigate and integrate AI effectively to optimize educational outcomes [42,43]. The acceptance of AI by teachers hinges on their understanding of its benefits and limitations, as well as institutional support for training and development [44]. For instance, AI can assist in creating personalized learning paths, automating administrative tasks, and providing data-driven insights that inform teaching strategies [46]. However, the success of AI in education depends not only on technological advancements but also on addressing ethical concerns, data privacy, and the potential impact on teacher employment [48,55]. Therefore, it is essential to foster a balanced approach that enhances the educational process while maintaining the irreplaceable human element in teaching [56]. Embracing this dual role of leveraging AI and preserving the core values of education can help teachers navigate the evolving landscape of modern classrooms effectively.

## 3. Aim and Importance of This Study

The current study seeks to answer the primary research question: “What is the level of awareness among teachers regarding the use of artificial intelligence (AI) in education?” To explore this question, we have defined the following sub-questions:

- Do the levels of awareness of AI in education vary according to the socio-demographic characteristics of teachers?
- Is the level of AI awareness among teachers influenced by their access to technology and the internet in education?
- Do teachers’ practical knowledge, beliefs, relatability, and theoretical knowledge of AI predict their level of AI awareness?

The questions of this research were determined in relation to the theoretical framework. The first question is related to the Technology Acceptance Model (TAM) and Rogers’ Theory of Diffusion of Innovation. The Technology Acceptance Model focuses on variables related to technology adoption. This research aims to investigate to what extent selected variables, such as age and gender, affect technology acceptance. Thus, the effectiveness of individual factors in technology acceptance was discussed. On the other hand, Rogers’ theory focuses on who are the early adopters and innovators in the life process. In this case, it is deemed worth researching to determine which socio-demographic characteristics people accept an innovation such as artificial intelligence. The second research question is about the impact of factors outside individuals. It was investigated whether environmental factors such as the internet technology used and access to technology affect teachers’ awareness of artificial intelligence. Answers were sought to questions such as whether these factors have an impact on the early adoption of artificial intelligence in relation to Rogers’ theory or whether they are effective in adopting technology as stated in the Technology Acceptance Model. Finally, the third research question is associated with Constructivist Learning Theory, which is based on the fact that acquiring knowledge is possible by doing and experiencing. In this question, the status of teachers’ practical and theoretical knowledge regarding artificial intelligence was investigated. Using AI technologies to “learn by doing” fosters positive views and practical knowledge, which in turn promotes increased awareness. This is consistent with Bloom’s Taxonomy, which promotes critical thinking and real-world application for thorough comprehension.

The aim of this study is to investigate the impact of teachers’ socio-demographic characteristics on their awareness of AI in education. Variables such as age, gender, education level, employment status in private or public schools, the educational levels at which they work (primary, secondary, tertiary, and university), and their salary/income levels were considered as independent variables. The primary objective is to determine how these socio-demographic factors influence teachers’ awareness of AI technologies and to use this information to make recommendations for educational policies and teacher training programs. This study thus aims to contribute to identifying the social and demographic factors necessary for more effective integration of AI in education.

The second dimension of this study focuses on analyzing the impact of teachers’ technology and internet usage habits in education on their AI awareness levels. This dimension is based on technology-focused questions. It was examined whether the search engines preferred by educators and the devices they use to connect to the internet affect their awareness of artificial intelligence. In addition, it was investigated whether the use of artificial intelligence in the course or which artificial intelligence tool was used affected the educators’ awareness of artificial intelligence. The aim is to reveal how much technology usage proficiency and diversity affects artificial intelligence awareness. As a result, teachers’ situations regarding educational technologies were discussed specifically in the context of artificial intelligence. It is thought that the findings will make it easier to create artificial intelligence education strategies and contribute to teachers’ adaptation to artificial intelligence applications in education.

In the last dimension of the research, teachers’ awareness levels of artificial intelligence in education were examined in the dimensions of practical knowledge, belief, relatability, and theoretical knowledge. This study aimed to provide a detailed description of teachers’ current understanding and practices of artificial intelligence technologies by examining them in four different dimensions. This comprehensive approach is important to optimize the efficient use of artificial intelligence in educational processes.

## 4. Materials and Methods

In the methods section of this study, the design of this study, the participants, the data collection tool, data collection procedures, and data analysis procedures were discussed.

Design: This study has been designed as a quantitative survey. This study is descriptive; it aims to depict teachers’ views on artificial intelligence as they exist. In this study, AI awareness among teachers is evaluated based on practical knowledge, belief-attitude, relatability, and theoretical knowledge. These components are crucial in understanding how teachers perceive and integrate AI into their teaching practices. The dependent variable of this study is the level of awareness concerning the use of artificial intelligence (AI); the independent variables are the socio-demographic characteristics of teachers (such as age, gender, seniority, and so on) presented in the objectives section.

Participants: In this study, teachers working at different educational levels in Northern Cyprus have been considered as research participants. According to the most recent statistical study conducted in Northern Cyprus, the total number of teachers working across all regions and levels was reported to be 5627 [64]. In this study, a purposive sampling and snowball technique was adopted, whereby researchers attempted to reach as many teachers as possible during the February to April period of the 2023–2024 academic year. This study focused on teachers who integrated AI into their lessons rather than those who did not. Random sampling was not an option in this instance to contact these teachers. Purposive and snowball sampling were therefore selected. Consequently, a total of 164 teachers participated in this study. The socio-demographic information of the teachers who participated in this study is presented in Table 1:

When the distribution of the teachers who participated in the artificial intelligence awareness level research according to age groups is analyzed, it is found that 51.8% of them are in the 22–29 age group, 17.7% are between 30 and 37 years old, 14.6% are between 38 and 45 years old, 7.9% are between 46 and 53 years old, and similarly 7.9% are between 54 years old and above. This study group was evenly distributed in terms of gender (50% in each group). In terms of education level, bachelor’s degree holders constitute the largest group with 72%, while the rate decreases as the level of education increases, where 20.1% are master’s degree holders and 7.9% are Ph.D. holders.

Sixty-one percent of the participants work in public schools and 39% in private schools, as shown in Table 2. When analyzed in terms of the educational level of the school where they work, 39.6% work in primary school, 18.3% in secondary school, 21.3% in high school, and 20.7% in university. When the monthly income levels are analyzed, it is seen that 47.6% of them receive a monthly salary between 24,000 and 30,000 TL, 15.2% between 31,000 and 37,000 TL, 21.3% between 38,000 and 44,000 TL, 7.3% between 45,000 and 51,000 TL, and 8.5% between 52,000 and more.

Data Collection Tool: In this study, a personal information form and the “Artificial Intelligence Awareness Scale” developed by Ferikoğlu and Akgün [25] were used. The personal information form consisted of two sections of multiple-choice questions; the first section contained socio-demographic questions (6 questions), and the second section included questions aimed at revealing computer and internet usage characteristics (4 questions). In both dimensions, the data were taken from teachers’ opinions. The validity and reliability of the Artificial Intelligence Awareness Scale were established by Ferikoğlu and Akgün; based on the results of the factor analysis, analyses can be conducted using the overall score of the scale as well as its four sub-dimensions (practical knowledge, belief-attitude, relatability, and theoretical knowledge). The scale, which consists of 51 items and uses a five-point Likert scale, has a Cronbach’s alpha of 0.986.

Data Collection Procedures: The data were collected over a three-month period from February to April 2024. The scale was prepared in an electronic format and distributed to teachers through school administrators. The snowball technique was utilized when sending the e-scale, and teachers were requested to forward the scale to their colleagues. An explanatory instruction was provided at the beginning of the scale, emphasizing voluntary participation, confidentiality, and other ethical considerations.

Data Analysis: Within the scope of this study, the data obtained from the teachers regarding the Artificial Intelligence Awareness Scale in the Northern part of Cyprus were analyzed with SPSS 24. The skewness and kurtosis values of all sub-dimensions of the Artificial Intelligence Awareness Scale were within the range of ±1.5 and met the normality condition, and the questions were tested with parametric statistics in this study (*t*-test, one-way ANOVA, and logistic regression). In addition, chi-square analysis was applied to compare the distribution of the use of AI according to school type and educational level. Multilayer Artificial Neural Network Analysis was performed to determine the degree of importance of the sub-dimensions of the Artificial Intelligence Awareness Scale on the use of artificial intelligence in teachers’ lessons.

## 5. Results

The findings section of this study is organized according to the research questions. Table 3 below provides the analysis of the data with regard to the first sub-question of this study.

The scores obtained from the scale of the teachers participating in the Artificial Intelligence Awareness Research in Northern Cyprus indicate that the mean score of the practical knowledge sub-dimension is $\bar{x}$ = 3.59, that is, above the average level. The mean score of beliefs and attitudes towards AI is $\bar{x}$ = 3.39, which is at a moderate level. The lowest mean score belongs to the relatability sub-dimension, and $\bar{x}$ = 3.36 is at a moderate level. The average score for theoretical knowledge ($\bar{x}$ = 3.45) is slightly above the middle level. The mean score for AI awareness of the participants was slightly above the middle level, $\bar{x}$ = 3.46. In addition, within the scope of this study, when the skewness and kurtosis scores for the normal distribution of the responses of the participants are examined, it is seen that (±1.5) the data are within the range suitable for normal distribution [65].

According to the results of this study, demographics such as age, gender, education level, type of school (public or private), institution level within the education system, and monthly income did not significantly affect the overall awareness of artificial intelligence or its sub-dimensions (*p* > 0.05). This indicates that these variables are not significant factors in the differing levels of AI awareness among teachers. The tables are presented as a Supplementary Materials.

The second research question aimed to determine whether the use of the internet and technology affects awareness of artificial intelligence. Findings from Table 4, Table 5, Table 6 and Table 7 address this question.

Among the teachers participating in this study, 45.7% of them stated that they used artificial intelligence in their lessons, while 54.3% stated that they did not. When the search engines they use are analyzed, it is seen that 85.4% of them use Google Chrome and 10.4% use Safari. Less than 5% used other search engines (1.2% Yandex, 1.2% Microsoft Bing, and 1.8% other search engines). When accessing the internet, 78.7% use their mobile phones, 13.4% use their laptops, 4.3% use their desktop computers, 3% use their tablets, and 0.6% use a different device. The most used artificial intelligence tool is ChatGPT, with 47%, while 39% use other tools, 4.3% use ChatON, 4.9% use Bing AL, and 4.9% use Replika.

There was no significant difference in the AI awareness levels or the sub-dimensions of the AI Awareness Scale among teachers who used AI in their lessons compared to those who did not (*p* > 0.05). Similarly, the type of AI tool used did not significantly affect the AI Awareness Scale outcomes (*p* > 0.05). Although the use of artificial intelligence was slightly more prevalent among teachers in private schools than in public schools, this difference was not statistically significant according to the chi-square test (*p* > 0.05). The tables are presented in the Supplementary Materials.

A significant difference was observed based on the search engine used by the teachers in the relatability dimension of AI, and the relatability of those using Microsoft Bing was higher than those using Google Chrome and Safari (*p* < 0.05). However, since the number of observations of Microsoft Bing users is low in this finding, this finding has limitations and should be supported with samples with higher numbers of search engine users. In other sub-dimensions, there was no significant difference in terms of search engine use (*p* > 0.05).

Although the theoretical knowledge dimension of the teachers showed a difference according to the type of device used, the theoretical knowledge level of those using laptops was higher than that of those using tablets (*p* < 0.05). However, it should be noted that this finding is limited due to the low number of observations of teachers using tablets. For this reason, this finding should be compared and supported with the findings of studies supported by more samples. In other sub-dimensions, no significant difference was observed in terms of the device used (*p* > 0.05).

In total, 52.3% of primary school teachers, 13.3% of middle school teachers, 40% of high school teachers, and 67.7% of university lecturers stated that they used AI in school lessons. The distribution of the above rates created a significant difference in the Chi-square test (*p* < 0.05). In the northern part of Cyprus, university lecturers were more likely to use AI in their lessons than other groups, followed by primary school teachers and then high school teachers. Secondary school teachers are the group that uses artificial intelligence the least in their lessons.

**Figure 1:**
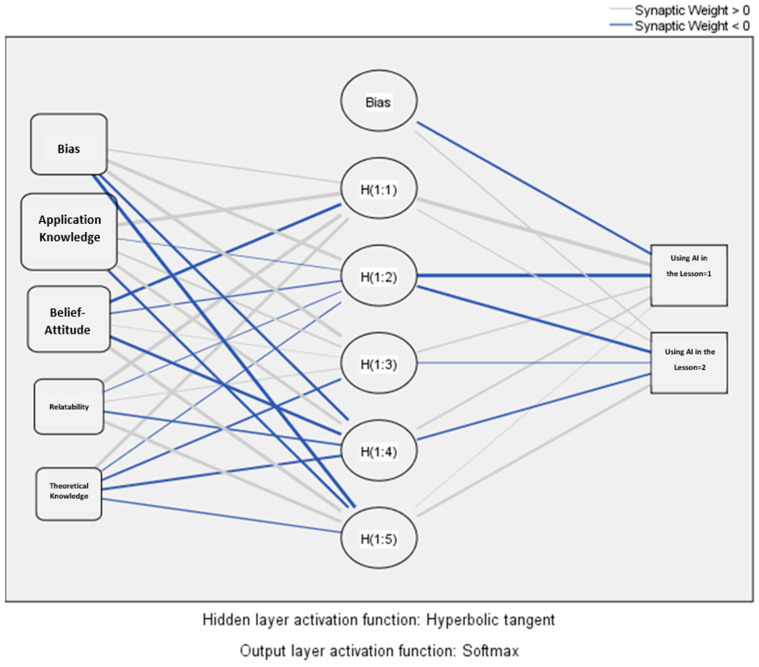
Multilayer Artificial Neural Network Analysis on the importance of the sub-dimensions of artificial intelligence applications scale in teachers’ use of artificial intelligence in their lessons.

**Figure 2:**
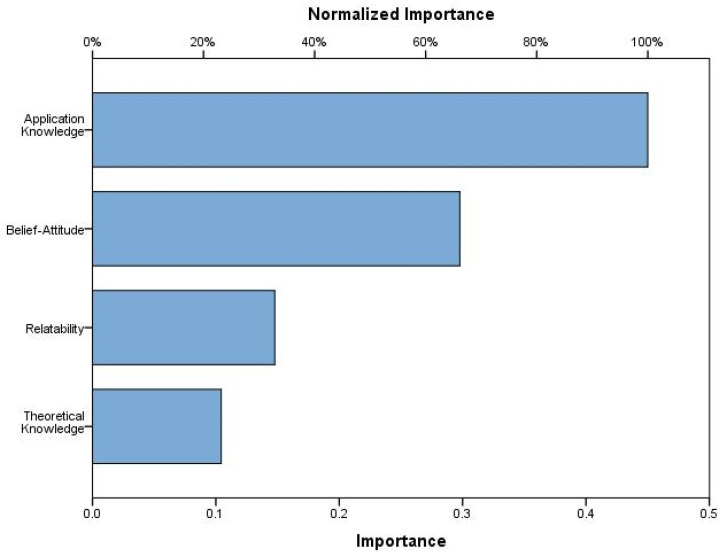
Importance level of scale sub-dimensions regarding teachers’ use of artificial intelligence in school lessons as a result of Multilayer Artificial Neural Network Analysis.

The third research question asked whether teachers’ practical knowledge, beliefs, relatability, and theoretical knowledge of AI predict their level of AI awareness. The findings are presented in Table 8 and Figure 1 and Figure 2.

According to the logistic regression results, implementation knowledge, beliefs-attitudes, relatability, and theoretical knowledge variables do not significantly predict the use of artificial intelligence in the lessons of the participants (*p* > 0.05).

As a result of the Multilayer Neural Network Analysis, it is seen that the most important influencing factor in teachers’ use of AI in lessons is practical knowledge (0.450, 100%). This is followed by beliefs and attitudes (0.298, 66.1%). Relatability is the third most important factor (0.148, 32.8%). Theoretical knowledge is the factor with the lowest importance (0.104, 23.2%). When these results are taken into consideration, it can be concluded that teachers should receive in-service trainings for practical application, as well as that the experiences they gain by applying artificial intelligence will be reflected in their beliefs and attitudes, and in this case, they should use this technology in their lessons.

The study’s findings, taken together, show that teachers in Northern Cyprus have an above-average awareness of AI, with practical knowledge ranking best and relatability ranking lowest. This study demonstrates that demographic variables, including age, gender, educational attainment, and kind of school, have no discernible impact on AI awareness. Furthermore, the choice of technical devices, especially laptops, had a substantial impact on theoretical understanding, but the usage of AI in the classroom by teachers and the kind of AI tools they employed had no significant effect on AI awareness. The study’s relationship to the theoretical framework demonstrates how well it aligns with constructivist learning theories, the Diffusion of Innovation Theory, and the Technology Acceptance Model (TAM). These frameworks emphasize the value of professional development and hands-on AI training, and they imply that teachers’ adoption and integration of AI in the classroom are greatly influenced by their practical knowledge and views. The results support the predictions of theoretical models of technology adoption and effective learning practices by highlighting the need for in-service training to improve practical application skills. These, in turn, can positively influence teachers’ attitudes and increase the use of AI in the classroom.

## 6. Discussion and Conclusions

This study provides significant insights into the current level of awareness among teachers in Northern Cyprus regarding the use of artificial intelligence (AI) in education. It highlights that while demographic factors such as age, gender, education level, and type of school do not significantly impact AI awareness, practical knowledge and attitudes towards AI play crucial roles in its integration into teaching practices. The findings emphasize the importance of practical AI training and professional development for educators to effectively utilize AI technologies in their classrooms. By fostering practical knowledge and positive attitudes, teachers can enhance their teaching strategies, customize learning experiences, and manage administrative tasks more efficiently, ultimately improving educational outcomes. This study underscores the need for targeted educational policies and training programs that equip teachers with the necessary skills and knowledge to leverage AI’s potential. Future research should explore longitudinal impacts and the role of specific educational policies in promoting AI integration in diverse educational settings.

The findings indicate that working in the public or private sector does not significantly affect educators’ awareness of using artificial intelligence (AI) in their teaching. This suggests that awareness levels may depend more on individual competencies or educational policies than the institutional environment. For instance, educators’ access to AI technologies and their confidence in using these technologies might be more decisive in influencing their awareness. Current AI research often emphasizes enhancing personalized learning and personalizing learning experiences [66,67]. The literature highlights the Technology Acceptance Model and the Diffusion of Innovation Theory, which underscore the role of individual attitudes and expectations in the process of adopting new technologies [26,27]. In this context, beyond the work environment, individual motivations and educational support systems could be more influential in the use of artificial intelligence.

The higher awareness of artificial intelligence (AI) usage among university faculty members, compared to other educational levels, can be attributed to factors such as academic freedom and access to resources. Universities typically have more resources available for research and innovation; thus, faculty members are more encouraged to explore and integrate new technologies. In higher education institutions, faculty members often engage in research-focused activities, which facilitate the use of innovative tools like AI as teaching materials [68]. This situation aligns with the group termed “early adopters” in Rogers’ Diffusion of Innovation Theory. Early adopters are more open to and inclined toward adopting innovative technologies [15].

The lack of significant impact from demographic factors such as age, gender, and educational level on the awareness of artificial intelligence (AI) usage might indicate the universality and broad accessibility of this technology. These findings suggest that the adoption of AI technologies is feasible, regardless of various demographic characteristics. According to the Technology Acceptance Model (TAM), perceived ease of use and perceived benefits in the technology adoption process may be more influential than demographic characteristics [28]. This implies that AI training programs conducted among different demographic groups could enhance general usage awareness. Educational and awareness-raising efforts are critical to disseminating this technology to a broader audience.

It has been observed among teachers that knowledge of artificial intelligence (AI) applications creates more awareness than theoretical knowledge alone. This suggests that practical applications may serve as a more effective tool for learning and engagement than theoretical instruction. Bloom’s Taxonomy and constructivist learning theories emphasize the importance of “learning by doing” in the learning process [29,30]. Practical applications enable the attainment of concrete results from theoretical knowledge and facilitate students’ adoption of new technologies. In this context, the integration of AI applications into curricula is seen to provide positive contributions to the learning process.

The findings of this study provide important insights into how studies on artificial intelligence (AI) education and awareness can be optimized. A better understanding of the underlying reasons and impacts of each finding could facilitate the more effective shaping of educational policies and strategies. According to research by Özer et al. [61], teachers need education and support first and foremost to effectively use AI tools. It is crucial that teachers possess the necessary knowledge and skills to effectively utilize AI in education. With the inclusion of AI into the educational process and tools, it is believed that the opportunities and innovations provided in the field of education will continue to increase [3]. Recent studies [58,62] suggest that teachers will increasingly move away from traditional education towards more intensive use of AI, making the educational process more active and productive.

This study highlights that while teachers in Northern Cyprus demonstrate a moderate to slightly above average awareness of artificial intelligence (AI), translating this awareness into educational practice remains a complex issue. The findings indicate that practical knowledge is the most significant factor influencing the use of AI in the classroom, followed by beliefs and attitudes, relatability, and theoretical knowledge. To effectively integrate AI into educational strategies, professional development programs should focus on enhancing teachers’ practical skills and providing hands-on experience with AI technologies. This approach is supported by current educational research, which emphasizes the importance of practice-oriented training in fostering positive attitudes and increased usage of technology in teaching [69,70].

Moreover, this study suggests that integrating AI into teaching practices can be further encouraged by addressing the specific needs and contexts of different educational levels. For instance, university lecturers were more likely to use AI in their lessons compared to primary and secondary school teachers, indicating that AI integration strategies might need to be tailored to different educational stages. Additionally, the usage patterns of AI tools reveal that there is a significant interest in and potential for AI applications in education. To harness this potential, it is crucial to develop targeted training programs that not only improve teachers’ technical skills but also align AI usage with pedagogical goals, ultimately enhancing teaching quality and learning outcomes [71,72]. By focusing on these areas, educational institutions can better support teachers in effectively integrating AI into their practices, thereby fostering a more innovative and effective learning environment.

The potential downsides of AI, such as issues of misuse and dependency, must be addressed to ensure the responsible integration of AI in educational settings. One significant concern is the development of problematic AI usage behaviors, which can be influenced by factors like academic self-efficacy, stress, and performance expectations [73]. To mitigate these risks, this study suggests implementing robust professional development programs that not only enhance teachers’ practical AI skills but also promote ethical usage and awareness of AI’s limitations. Educators should be equipped with strategies to balance the benefits of AI tools with mindfulness about potential over-reliance, ensuring that AI augments rather than diminishes the educational experience. Moreover, integrating AI literacy into the curriculum can help students understand both the capabilities and risks of AI, fostering a generation of informed and responsible AI users [74,75]. These steps can help mitigate the negative impacts while maximizing the positive educational outcomes of AI technology.

Limitations of this study include its cross-sectional design, lack of detailed measurement of awareness, and potential variability in technological access, which could impact the findings. Owing to the extensive number of dimensions and the multitude of factors examined in the research, the variables that failed to demonstrate a statistically significant difference were not examined. Subsequent investigations ought to concentrate on long-term studies, examine the consequences of particular educational policies, and examine the function of technology integration models in the educational process. Future studies should look into the possible drawbacks of AI that were covered in the results and consult with educators to learn about their thoughts on these matters. Recommendations for enhancing AI integration include providing professional development focusing on both theoretical and practical applications of AI, integrating AI into curricula, ensuring equitable resource allocation across institutions, establishing support systems for educators, and tailoring programs to diverse demographic needs to promote effective and inclusive AI education.

## Figures and Tables

**Table 1:** Demographic characteristics of teachers participating in the artificial intelligence awareness level research.

		f	%
Age	22–29	85	51.8
	30–37	29	17.7
	38–45	24	14.6
	46–53	13	7.9
	54+	13	7.9
Gender	Female	82	50.0
	Male	82	50.0
Education level	Bachelor’s	118	72.0
	Master’s degree	33	20.1
	Ph.D.	13	7.9
	Total	164	100.0

**Table 2:** Job type and income status of teachers participating in artificial intelligence awareness research.

		f	%
School type	Public school	100	61.0
	Private school	64	39.0
Level of the educational institution	Primary school	65	39.6
	Middle school	30	18.3
	High school	35	21.3
	University	34	20.7
Monthly income level	24,000–30,000 TL	78	47.6
	31,000–37,000 TL	25	15.2
	38,000–44,000 TL	35	21.3
	45,000–51,000 TL	12	7.3
	52,000 TL+	14	8.5
	Total	164	100.0

TL: Turkish liras.

**Table 3:** Descriptive analysis for the Artificial Intelligence Awareness Scale for teachers’ sub-dimensions and overall.

	N	Min	Max	$\bar{x}$	Σ	Skewness	Kurtosis
Practical knowledge	164	1.00	5.00	3.59	1.01	−1.257	0.758
Belief-attitude	164	1.00	5.00	3.39	0.959	−0.902	0.424
Relatability	164	1.00	5.00	3.36	0.931	−0.815	0.510
Theoretical knowledge	164	1.00	5.00	3.45	0.995	−1.117	0.592
Artificial intelligence awareness	164	1.00	5.00	3.46	0.945	−1.160	0.891

**Table 4:** Artificial intelligence awareness, use of artificial intelligence courses, and habits of using technology.

		f	%
Using artificial intelligence for course	Yes	75	45.7
	No	89	54.3
Search engine used	Google Chrome	140	85.4
	Safari	17	10.4
	Yandex	2	1.2
	Microsoft Bing	2	1.2
	Other	3	1.8
The most used device for internet access	Mobile phone	129	78.7
	Laptop	22	13.4
	Desktop computer	7	4.3
	Tablet	5	3.0
	Other	1	0.6
The most used artificial intelligence tool	ChatGPT	77	47.0
	ChatOn	7	4.3
	Bing AL	8	4.9
	Replika	8	4.9
	Other	64	39.0
	Total	164	100.0

**Table 5:** Comparison of the Artificial Intelligence Awareness Scale and its sub-dimensions in terms of search engine usage.

	Group	N	$\bar{x}$	σ		Sum of Squares	F	*p*
Relatability	Google Chrome	140	3.38	0.899	Between groups	8.899	2.672	0.034
	Safari	17	2.94	0.961	Within groups	132.389		
	Yandex	2	3.75	0.353	Total	141.288		
	Microsoft Bing	2	5.00	0.000				
	Other	3	3.10	1.58				
	Other	3	3.17	1.64				

**Table 6:** Comparison of the Artificial Intelligence Awareness Scale and its sub-dimensions in terms of technological device use.

	Group	N	$\bar{x}$	Σ		Sum of Squares	F	*p*
Theoretical knowledge	Mobile phone	129	3.41	0.988	Between groups	11.182	2.958	0.022
	Laptop	22	3.90	0.719	Within groups	150.253		
	Desktop computer	7	3.41	1.01	Total	161.435		
	Tablet	5	2.32	1.426				
	Other	1	3.72	.				

**Table 7:** Teachers’ use of artificial intelligence in school lessons according to the education level of the institution where they work.

			Level of the Educational Institution				Total			
			Primary School	Middle School	High School	University		χ2	df	*p*
Use of artificial intelligence for school courses	Yes	*f*	34	4	14	23	75	20.864 ^a^	3	0.000
		%	52.3%	13.3%	40.0%	67.6%	45.7%			
	No	*f*	31	26	21	11	89			
		%	47.7%	86.7%	60.0%	32.4%	54.3%			
Total		*f*	65	30	35	34	164			
		%	100.0%	100.0%	100.0%	100.0%	100.0%			

a. 0 cells (.0%) have expected count less than 5. The minimum expected count is 13.72.

**Table 8:** Logistic regression analysis results for the prediction of teachers’ practical knowledge, belief-attitude, relatability, and theoretical knowledge about artificial intelligence on the use of artificial intelligence in their lessons.

		Exp (B)	B	S.E.	Wald	df	*p*
Step 1 ^a^	Practical knowledge	0.536	−0.623	0.444	1.965	1	0.161
	Belief-attitude	2.110	0.747	0.497	2.256	1	0.133
	Relatability	0.647	−0.436	0.527	0.683	1	0.408
	Theoretical knowledge	1.243	0.217	0.500	0.189	1	0.664
	Constant	1.812	0.594	0.613	0.940	1	0.332

a. It refers to the independent variables included in the logistic regression model.

## Data Availability

The raw data supporting the conclusions of this article will be made available by the authors without undue reservation.

## Supplementary Materials

The following supporting information can be downloaded at: https://www.mdpi.com/article/10.3390/ejihpe14080156/s1, Table S1: Results showing no significant difference from the statistical analysis.
