# Supplementary material for: Exploring Teacher Awareness of Artificial Intelligence in Education: A Case Study from Northern Cyprus
Source: Eur J Investig Health Psychol Educ. 2024 Aug 12;14(8):2358–76. doi: 10.3390/ejihpe14080156 (PMC11354280; doi:10.3390/ejihpe14080156)
Supplement: Supplementary file 1 [file ejihpe-14-00156-s001.zip › ejihpe-3090429-supplementary.pdf]

Results showing no significant difference from the statistical analysis

Table. Comparison of Teachers' Use of Artificial Intelligence for School Course According to School Type

			School Type					
			Public	Private	Total	$\chi 2$	df	p
Use of Artificial Intelligence for School Course	Yes	*f*	44	31	75	.310[a]	1	.578
		%	44.0%	48.4%	45.7%			
	No	*f*	56	33	89			
		%	56.0%	51.6%	54.3%			
Total		*f*	100	64	164			
		%	100.0%	100.0%	100.0%			

While 44% of teachers working in public schools stated that they use artificial intelligence in their school lessons, 48.4% of teachers working in private schools stated that they use artificial intelligence in their lessons. While 56% of public school teachers do not use artificial intelligence in their lessons, 51.6% of private school teachers do not. Although the rate of teachers working in private schools using artificial intelligence in their lessons is slightly higher than those working in public schools, it did not create a significant difference according to the chi-square test (p>0.05).

Table. Comparison of Artificial Intelligence Awareness Scale and Sub-dimensions of Teachers in the North of Cyprus According to Age Groups

	Group	N	$\bar{x}$	σ		Sum of Squares	F	p
Application Knowledge	22-29	85	3.68	1.02	Between Groups	5.424	1.323	.264
	30-37	29	3.43	1.05	Within Groups	162.939		
	38-45	24	3.71	.815	Total	168.363		
	46-53	13	3.69	.952				
	54+	13	3.06	1.21				
Belief-Attitude	22-29	85	3.47	.943	Between Groups	6.043	1.668	.160
	30-37	29	3.36	1.02	Within Groups	143.982		
	38-45	24	3.41	.823	Total	150.025		
	46-53	13	3.50	1.02				
	54+	13	2.75	.981				
Relatability	22-29	85	3.46	.922	Between Groups	6.990	2.069	.087
	30-37	29	3.27	1.02	Within Groups	134.298		
	38-45	24	3.35	.736	Total	141.288		
	46-53	13	3.52	1.02				
	54+	13	2.71	.850				
Theoretical Knowledge	22-29	85	3.57	.983	Between Groups	6.430	1.649	.165
	30-37	29	3.26	1.05	Within Groups	155.005		
	38-45	24	3.45	.769	Total	161.435		
	46-53	13	3.58	1.02				
	54+	13	2.90	1.15				
Artificial Intelligence Awareness	22-29	85	3.56	.935	Between Groups	5.867	1.669	.160
	30-37	29	3.34	1.00	Within Groups	139.707		
	38-45	24	3.50	.770	Total	145.574		
	46-53	13	3.58	.968				
	54+	13	2.87	1.031				

There was no significant difference between the age groups in the overall AI awareness levels of the teachers in the Northern part of Cyprus and in the sub-dimensions of the scale (p>0.05).

Table. Comparison of Artificial Intelligence Awareness Scale and Sub-dimensions of Teachers in the Northern part of Cyprus by Gender

	Group	N	$\bar{x}$	σ	t	*df*	p
Application Knowledge	Female	82	3.59	.961	.053	162	.958
	Male	82	3.59	1.07			
Belief-Attitude	Female	82	3.42	.922	.412	162	.681
	Male	82	3.36	.999			
Relatability	Female	82	3.32	.901	-.460	162	.646
	Male	82	3.39	.964			
Theoretical Knowledge	Female	82	3.43	.959	-.256	162	.798
	Male	82	3.47	1.03			
Artificial Intelligence Awareness	Female	82	3.46	.905	-.015	162	.988
	Male	82	3.46	.988			

It was observed that there was no difference in terms of gender in the overall level of awareness of Artificial Intelligence among Teachers in the North of Cyprus and in the sub-dimensions of the scale, and in this context, gender is not a variable that will make a difference in this regard (p>0.05).

Table. Comparison of Artificial Intelligence Awareness Scale and Sub-dimensions of Teachers in the North of Cyprus According to Teachers' Level of Education

	Group	N	$\bar{x}$	σ		Sum of Squares	F	p
Application Knowledge	Bachelor's	118	3.53	1.06	Between Groups	1.652	.798	.452
	Master's Degree	33	3.74	.751	Within Groups	166.711		
	PhD	13	3.78	1.17	Total	168.363		
Belief-Attitude	Bachelor's	118	3.33	.962	Between Groups	1.541	.835	.436
	Master's Degree	33	3.56	.794	Within Groups	148.485		
	PhD	13	3.51	1.28	Total	150.025		
Relatability	Bachelor's	118	3.32	.941	Between Groups	.549	.314	.731
	Master's Degree	33	3.46	.737	Within Groups	140.739		
	PhD	13	3.41	1.27	Total	141.288		
Theoretical Knowledge	Bachelor's	118	3.41	1.01	Between Groups	.567	.284	.753
	Master's Degree	33	3.53	.838	Within Groups	160.868		
	PhD	13	3.56	1.25	Total	161.435		
Artificial Intelligence Awareness	Bachelor's	118	3.41	.966	Between Groups	1.085	.605	.547
	Master's Degree	33	3.59	.738	Within Groups	144.488		
	PhD	13	3.59	1.21	Total	145.574		

It was observed that the level of education of the teachers in the North of Cyprus did not create a significant difference in terms of artificial intelligence awareness and the sub-dimensions of this scale (p>0.05).

Table. Comparison of Artificial Intelligence Awareness Scale and Sub-dimensions of Teachers in Northern Cyprus According to Public and Private Schools

	Group	N	x̅	σ	t	*df*	p
Application Knowledge	Public School	100	3.66	.911	1.143	162	.255
	Private School	64	3.48	1.15			
Belief-Attitude	Public School	100	3.47	.86	1.316	162	.190
	Private School	64	3.27	1.09			
Relatability	Public School	100	3.38	.82	.367	162	.714
	Private School	64	3.32	1.08			
Theoretical Knowledge	Public School	100	3.52	.90	1.228	162	.221
	Private School	64	3.33	1.11			
Artificial Intelligence Awareness	Public School	100	3.52	.84	1.101	162	.272
	Private School	64	3.36	1.08			

There was no difference between teachers working in public and private schools in the Northern part of Cyprus in terms of the artificial intelligence awareness scale and its sub-dimensions (p>0.05).

Table. Comparison of Artificial Intelligence Awareness Scale and Sub-Dimensions of Teachers According to the Level of the Educational Institution They Work in

	Group	N	x̅	σ		Sum of Squares	F	p
Application Knowledge	Primary School	65	3.51	1.05	Between Groups	1.362	.435	.728
	Middle School	30	3.71	.71	Within Groups	167.001		
	High School	35	3.53	1.13	Total	168.363		
	University	34	3.70	1.05				
Belief-Attitude	Primary School	65	3.35	.99	Between Groups	1.700	.611	.609
	Middle School	30	3.56	.67	Within Groups	148.325		
	High School	35	3.25	1.07	Total	150.025		
	University	34	3.45	.99				
Relatability	Primary School	65	3.31	.95	Between Groups	1.795	.686	.562
	Middle School	30	3.49	.67	Within Groups	139.493		
	High School	35	3.22	1.02	Total	141.288		
	University	34	3.48	.988				
Theoretical Knowledge	Primary School	65	3.47	1.02	Between Groups	1.266	.422	.738
	Middle School	30	3.51	.73	Within Groups	160.169		
	High School	35	3.28	1.11	Total	161.435		
	University	34	3.51	1.04				
Artificial Intelligence Awareness	Primary School	65	3.42	.97	Between Groups	1.315	.486	.692
	Middle School	30	3.58	.65	Within Groups	144.258		
	High School	35	3.34	1.06	Total	145.574		
	University	34	3.55	.98				

There was no significant difference in the overall artificial intelligence awareness and the sub-dimensions of this scale according to the level of the institution where the teachers in Northern Cyprus work within the education system (p>0.05).

Table. Comparison of Artificial Intelligence Awareness Scale and Sub-dimensions of Teachers in the North of Cyprus According to Monthly Income

	Group	N	$\bar{x}$	σ		Sum of Squares	F	p
Application Knowledge	24.000-30.000 TL	78	3.49	1.11	Between Groups	2.123	.508	.730
	31.000-37.000 TL	25	3.64	1.01	Within Groups	166.240		
	38.000-44.000 TL	35	3.77	.640	Total	168.363		
	45.000-51.000 TL	12	3.57	1.12				
	52.000 TL+	14	3.66	1.17				
Belief-Attitude	24.000-30.000 TL	78	3.27	1.06	Between Groups	3.386	.918	.455
	31.000-37.000 TL	25	3.36	.91	Within Groups	146.639		
	38.000-44.000 TL	35	3.64	.52	Total	150.025		
	45.000-51.000 TL	12	3.38	1.00				
	52.000 TL+	14	3.45	1.21				
Relatability	24.000-30.000 TL	78	3.31	1.00	Between Groups	.682	.193	.942
	31.000-37.000 TL	25	3.32	.87	Within Groups	140.606		
	38.000-44.000 TL	35	3.47	.60	Total	141.288		
	45.000-51.000 TL	12	3.37	1.13				
	52.000 TL+	14	3.38	1.18				
Theoretical Knowledge	24.000-30.000 TL	78	3.41	1.05	Between Groups	.842	.208	.933
	31.000-37.000 TL	25	3.41	.977	Within Groups	160.593		
	38.000-44.000 TL	35	3.55	.72	Total	161.435		
	45.000-51.000 TL	12	3.31	1.17				
	52.000 TL+	14	3.55	1.18				
Artificial Intelligence Awareness	24.000-30.000 TL	78	3.38	1.03	Between Groups	1.621	.448	.774
	31.000-37.000 TL	25	3.45	.91	Within Groups	143.953		
	38.000-44.000 TL	35	3.63	.57	Total	145.574		
	45.000-51.000 TL	12	3.42	1.07				
	52.000 TL+	14	3.52	1.15				

There were no significant differences between the monthly income levels of the teachers in the Northern part of Cyprus in the overall AI awareness scale and its sub-dimensions (p>0.05).

Table. Comparison of Artificial Intelligence Awareness Scale and its Sub-dimensions According to the Use of Artificial Intelligence Awareness Scale and its Sub-dimensions in School Lessons by Teachers in the North of Cyprus

	Group	N	$\bar{x}$	σ	t	*df*	p
Application Knowledge	Yes	75	3.67	1.00	.879	162	.381
	No	89	3.53	1.02			
Belief-Attitude	Yes	75	3.41	.95	.234	162	.815
	No	89	3.37	.96			
Relatability	Yes	75	3.41	.95	.686	162	.494
	No	89	3.31	.91			
Theoretical Knowledge	Yes	75	3.49	.98	.567	162	.572
	No	89	3.40	1.00			
Artificial Intelligence Awareness	Yes	75	3.51	.94	.623	162	.534
	No	89	3.42	.947			

There was no significant difference in the AI awareness levels and AI awareness levels scale sub-dimensions of the teachers who used and did not use Artificial Intelligence in their lessons in the Northern part of Cyprus (p>0.05).

Table. Comparison of Artificial Intelligence Awareness Scale And Its Sub-Dimensions In Terms of Search Engine Usage

	Group	N	$\bar{x}$	σ		Sum of Squares	F	p
Application Knowledge	Google Chrome	140	3.61	.98	Between Groups	5.887	1.440	.223
	Safari	17	3.29	1.12	Within Groups	162.475		
	Yandex	2	3.62	.441	Total	168.363		
	Microsoft Bing	2	5.00	.000				
	Diğer	3	3.25	1.69				
Belief-Attitude	Google Chrome	140	3.41	.936	Between Groups	8.266	2.318	.059
	Safari	17	3.05	.97	Within Groups	141.759		
	Yandex	2	3.92	.10	Total	150.025		
	Microsoft Bing	2	5.00	.00				
	Other	3	2.97	1.53				
Relatability	Google Chrome	140	3.38	.899	Between Groups	8.899	2.672	.034
	Safari	17	2.94	.961	Within Groups	132.389		
	Yandex	2	3.75	.353	Total	141.288		
	Microsoft Bing	2	5.00	.000				
	Other	3	3.10	1.58				
Theoretical Knowledge	Google Chrome	140	3.46	.958	Between Groups	6.774	1.741	.144
	Safari	17	3.11	1.138	Within Groups	154.661		
	Yandex	2	4.00	.000	Total	161.435		
	Microsoft Bing	2	4.90	.128				
	Other	3	3.39	1.77				
Artificial Intelligence Awareness	Google Chrome	140	3.48	.915	Between Groups	7.118	2.044	.091
	Safari	17	3.12	1.01	Within Groups	138.456		
	Yandex	2	3.81	.041	Total	145.574		
	Microsoft Bing	2	4.98	.027				
	Other	3	3.17	1.64				

A significant difference was observed according to the search engine used by the teachers in the theoretical knowledge dimension of artificial intelligence, and the theoretical knowledge level of those using Microsoft bing was higher than those using Google Chrome and Safari (p<0.05). However, since the number of observations of Microsoft bing users is low in this finding, this finding has limitations and should be supported with samples with higher numbers of search engine users. In other sub-dimensions, there was no significant difference in terms of search engine use (p>0.05).

Table. Comparison of Artificial İntelligence Awareness Scale And İts Sub-Dimensions In Terms of Technological Device Use

	Group	N	$\bar{x}$	σ		Sum of Squares	F	p
Application Knowledge	Mobile Phone	129	3.55	1.02	Between Groups	6.668	1.639	.167
	Laptop	22	3.96	.728	Within Groups	161.695		
	Desktop Computer	7	3.70	.927	Total	168.363		
	Tablet	5	2.81	1.60				
	Other	1	4.06	.				
Belief-Attitude	Mobile Phone	129	3.36	.980	Between Groups	4.060	1.106	.356
	Laptop	22	3.72	.702	Within Groups	145.965		
	Desktop Computer	7	3.27	.887	Total	150.025		
	Tablet	5	2.85	1.39				
	Other	1	3.50	.				
Relatability	Mobile Phone	129	3.34	.955	Between Groups	4.954	1.444	.222
	Laptop	22	3.66	.720	Within Groups	136.334		
	Desktop Computer	7	3.20	.673	Total	141.288		
	Tablet	5	2.66	1.20				
	Other	1	3.80	.				
Theoretical Knowledge	Mobile Phone	129	3.41	.988	Between Groups	11.182	2.958	.022
	Laptop	22	3.90	.719	Within Groups	150.253		
	Desktop Computer	7	3.41	1.01	Total	161.435		
	Tablet	5	2.32	1.426				
	Other	1	3.72	.				
Artificial Intelligence Awareness	Mobile Phone	129	3.43	.958	Between Groups	6.199	1.768	.138
	Laptop	22	3.82	.666	Within Groups	139.375		
	Desktop Computer	7	3.42	.852	Total	145.574		
	Tablet	5	2.69	1.41				
	Other	1	3.78	.				

Although the theoretical knowledge dimension of the teachers showed a difference according to the type of device used, the theoretical knowledge level of those using laptops was higher than those using tables ($p<0.05$). However, it should be noted that this finding is limited due to the low number of observations of teachers using tablets. For this reason, this finding should be compared and supported with the findings of studies supported by more samples. In other sub-dimensions, no significant difference was observed in terms of the device used ($p>0.05$).

Table. Comparison of Artificial Intelligence Awareness Scale and Its Subscales in Terms of Artificial Intelligence Tool Use

	Group	N	$\bar{x}$	σ		Sum of Squares	F	p
Application Knowledge	ChatGPT	77	3.67	.957	Between Groups	8.635	2.149	.077
	ChatOn	7	2.81	1.393	Within Groups	159.728		
	Bing AL	8	4.21	.657	Total	168.363		
	Replika	8	3.34	1.178				
	Other	64	3.53	1.021				
Belief-Attitude	ChatGPT	77	3.41	.940	Between Groups	4.579	1.251	.291
	ChatOn	7	2.94	1.16	Within Groups	145.446		
	Bing AL	8	4.00	.711	Total	150.025		
	Replika	8	3.28	1.035				
	Other	64	3.35	.967				
Relatability	ChatGPT	77	3.3442	.884	Between Groups	3.445	.993	.413
	ChatOn	7	3.1571	1.28	Within Groups	137.843		
	Bing AL	8	3.9500	.834	Total	141.288		
	Replika	8	3.1500	1.05				
	Other	64	3.3578	.940				
Theoretical Knowledge	ChatGPT	77	3.43	.932	Between Groups	4.318	1.093	.362
	ChatOn	7	3.02	1.45	Within Groups	157.116		
	Bing AL	8	3.98	.691	Total	161.435		
	Replika	8	3.15	1.27				
	Other	64	3.47	1.00				
Artificial Intelligence Awareness	ChatGPT	77	3.48	.894	Between Groups	5.018	1.419	.230
	ChatOn	7	2.96	1.28	Within Groups	140.555		
	Bing AL	8	4.05	.684	Total	145.574		
	Replika	8	3.25	1.12				
	Other	64	3.43	.958				

It is seen that teachers' Artificial Intelligence Awareness Scale and its sub-dimensions do not show a significant difference according to the AI tool used ($p>0.05$).
